# Vanishing native American dog lineages

**DOI:** 10.1186/1471-2148-11-73

**Published:** 2011-03-21

**Authors:** Santiago Castroviejo-Fisher, Pontus Skoglund, Raúl Valadez, Carles Vilà, Jennifer A Leonard

**Affiliations:** 1 Department of Evolutionary Biology, Uppsala University, 75236 Uppsala, Sweden; 2 Departamento de Ciencias Biológicas, Universidad de los Andes, Bogota, Colombia; 3 Department of Herpetology, American Museum of Natural History, Central Park West at 79th Street, New York, NY 10024-5192, USA; 4 Laboratorio de Paleozoología, Instituto de Investigaciones Antropológicas, Universidad Nacional Autónoma de México. Circuito Exterior, Ciudad Universitaria, C.P. 04510, Distrito Federal, México; 5 Conservation and Evolutionary Genetics Group, Estación Biológica de Doñana (EBD-CSIC), Avd. Américo Vespucio s/n, 41092 Seville, Spain

## Abstract

**Background:**

Dogs were an important element in many native American cultures at the time Europeans arrived. Although previous ancient DNA studies revealed the existence of unique native American mitochondrial sequences, these have not been found in modern dogs, mainly purebred, studied so far.

**Results:**

We identified many previously undescribed mitochondrial control region sequences in 400 dogs from rural and isolated areas as well as street dogs from across the Americas. However, sequences of native American origin proved to be exceedingly rare, and we estimate that the native population contributed only a minor fraction of the gene pool that constitutes the modern population.

**Conclusions:**

The high number of previously unidentified haplotypes in our sample suggests that a lot of unsampled genetic variation exists in non-breed dogs. Our results also suggest that the arrival of European colonists to the Americas may have led to an extensive replacement of the native American dog population by the dogs of the invaders.

## Background

Dogs colonized the Americas with early human groups from Asia [1] and were widespread by the time Europeans arrived late in the 15th century [2]. Most of the dogs around the world today have mitochondrial DNA (mtDNA) control region sequences that form a well-defined phylogenetic clade (Clade I) [3]. Genetic characterization of ancient American dogs (samples obtained from human settlements that had not been in contact with Europeans, hereafter referred to as native American dogs) revealed a unique set of mtDNA sequences that clustered within this clade, but have not been observed in extant dogs [*e.g*. [3-6]]. Most notably, a subclade (I*a*) has so far only been identified in ancient American dogs and had a frequency of 62% in ancient Latin American animals [1]. However, most genetic studies are based on purebred dogs, and since most internationally recognized breeds today are primarily European or Asian in origin, it is possible that American dog lineages have been excluded.

**Figure 1:**
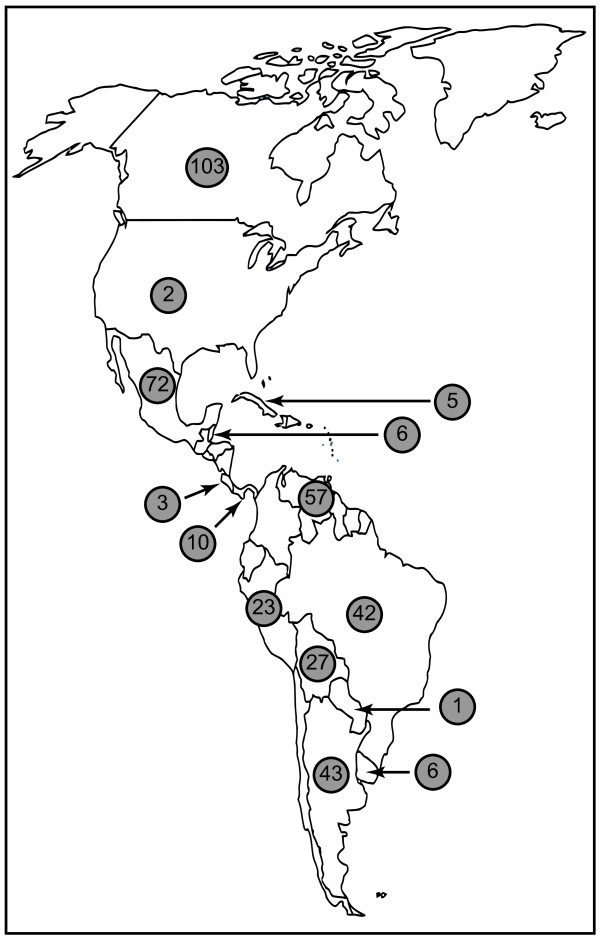
**Map illustrating geographic distribution of modern American dog samples included in the study**. Numbers indicate number sample size per country.

To determine contemporary distribution and frequency of the native American dog lineages in the Americas (both North America and South America), we analyzed the fragment of the mtDNA control region comparable to available genetic data for ancient native American dogs in 400 village and non-breed dogs from Alaska to Patagonia. These included dogs living in small isolated settlements in Canada, Mexico, Central America, the Caribbean, the Orinoco Llanos, the Andes, the Amazon basin and Patagonia (Figure 1). This novel data set allowed us to compare the genetic composition of past and present populations, and to use statistical population genetic models to estimate the maximum possible contribution of pre-Columbian dogs to the extant population.

## Results and discussion

In the total sample of 400 modern American dogs (Genbank numbers in Table S1), we identified 40 unique mtDNA haplotypes, of which 23 (57.5%) had not been identified in previous studies that included samples from around the world. Haplotypes were widespread across sampled localities and we did not detect any geographic pattern. This shows that significant undescribed diversity is likely to be present in non-purebred dogs [5]. In addition, the level of mtDNA nucleotide diversity in the present Latin American population (θ_π _= 0.017,  = 40,000) indicates an effective population size similar to that in ancient America (θ_π _= 0.015,  = 35,000). 

**Figure 2:**
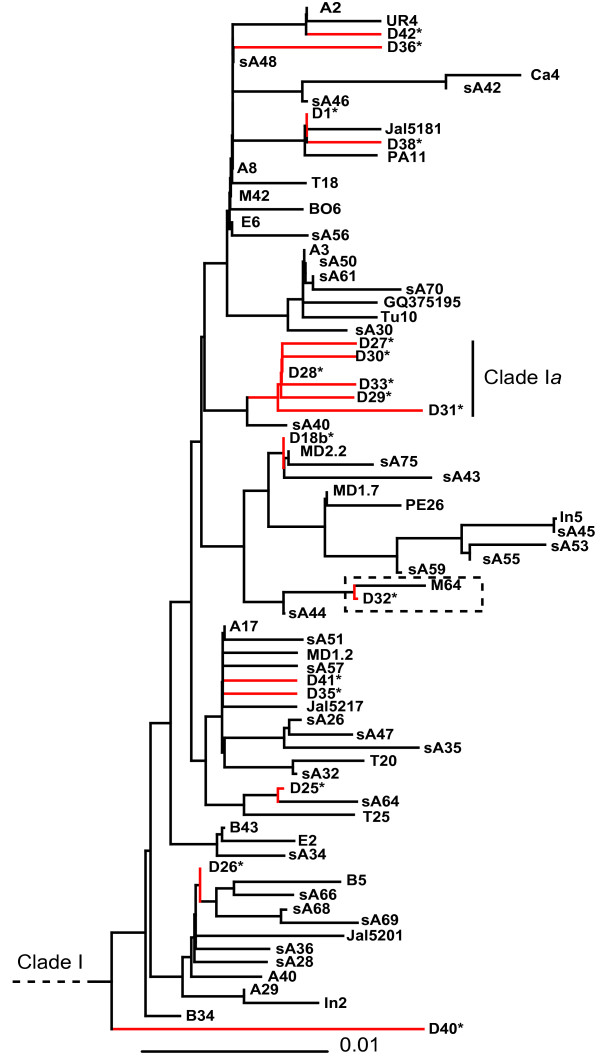
**Maximum likelihood phylogeny of Clade I haplotypes**. Ancient American dog haplotypes are on red branches. Clade I*a *is highlighted with a label and the dashed rectangle highlights the only extant haplotype inferred to be derived from an ancient dog sequence. Tip labels correspond to non-redundant haplotypes from Table S1 or [1,3-5,7].

More than half of the haplotypes (23/40) and individuals (259/400) belonged to the common Clade I. However, none of the sequences was identical to nor clustered within the ancient native American dog Clade I*a *(Figure 2). This striking difference in haplogroup frequencies between ancient and modern dogs suggests that very little of the mtDNAs present in the extant population trace back to the native American population This could be due to either genetic drift or population discontinuity between the two time points.

We investigated the probability of this result under different demographic models and found that the observation of a private haplogroup in the ancient sample with a frequency as high as I*a *allows rejection of complete continuity between ancient and present populations for an extensive range of assumptions. For the constant size population model (see Methods), we found that all models with an assumed *N_e _*> 3,000 could be rejected at the 5% significance level, and the population expansion scenario was similarly rejected for initial *N_e _*> 2,100 (P < 0.05).

**Figure 3:**
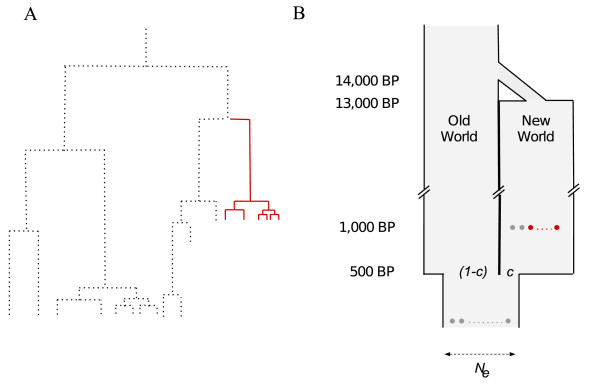
**Coalescent simulations used to investigate the maximum possible genetic contribution of the native American dog population to the living American dog population**. A) From genealogies generated under three different models, we estimated the probability that at least 8 of 13 ancient lineages coalesce to the exclusion of all modern samples. B) Schematic illustration of the isolation-admixture model.

**Figure 4:**
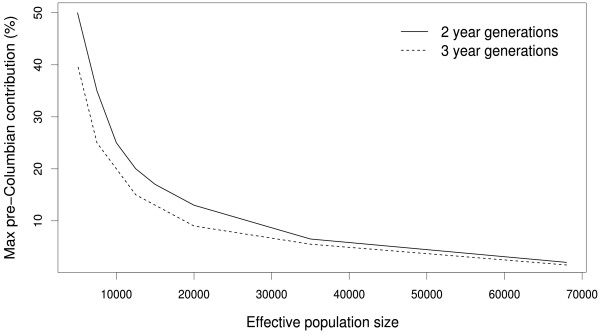
**Maximum genetic contribution of native American dogs under the isolation-admixture model**. Lines represent the 95 percent limit under different assumptions about effective population size (varying across the x-axis) and generation time. The results assuming a two year generation time are shown with a solid curve and the results assuming a three year generation time with a dashed curve.

However, complete continuity is not a realistic model since introduction of European dogs is known to have occurred. Thus, we also tested the possible contribution of the ancient population under a model where the current Latin American dog population is a mixture of Old- and New World populations (Figure 3). The simulations indicated that given the estimated mitochondrial , less than 10% of the ancestors of the modern American dog population are ancient American dogs (Figure 4). While based on inferences from a single genetic marker, this upper limit for the average genetic contribution of the ancient population is expected to apply also to the autosomal genomes of extant dogs. However, we caution that the average genome-wide native American ancestry in the extant population could be different if male and female dogs contributed an unequal number of offspring to the population (*i.e*. there was an unequal sex ratio), the mitochondrion has been subject to selection, or if other assumptions in the demographic model (see Methods) have been violated in the recent history of the population.

**Figure 5:**
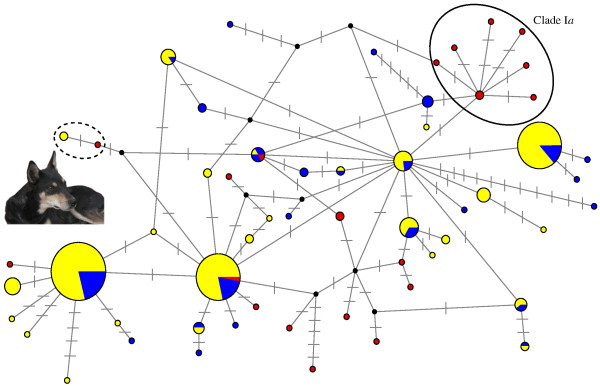
**Median joining network of Clade I haplotypes**. Solid ellipse highlights Clade I*a *and dashed ellipse highlights the only extant haplotype inferred to be derived from an ancient American dog haplotype and that ancient haplotype. Node size is proportional to haplotype frequency. Transversal lines indicate mutations and black dots hypothetical haplotypes. Ancient American haplotypes are red, contemporary American haplotypes are yellow and contemporary dogs from elsewhere in the world are blue. Photograph inset: dog from Chan Kom (M76 in Table S1), a likely descendant of pre-Columbian native American dogs.

We also examined if ancient American haplotypes other than those in Clade I*a *were present in the modern sample, and identified a single modern haplotype derived from a pre-Columbian haplotype (Figure 5). This previously undescribed haplotype was found in two dogs from the Maya villages of Pisté and Chan Kom, both in the State of Yucatán, Mexico (M64, M76; Additional file 1: Table S1) and is most closely related to the ancient Mexican haplotype D32 [1], from which it differed by a single substitution. Although these communities are in close contact with modern life, they still live largely according to indigenous traditions, including a lack of organized dog husbandry. The mtDNA of these two individuals may be inherited from the pre-Columbian population, and thus indicate that not all ancient lineages went completely extinct.

Arrival by Columbus to the Americas in 1492 was quickly followed by the arrival of conquistadors, missionaries and colonists from Europe with their livestock, pets, commensals and pathogens, all of which had an important impact on native American populations and culture [7,8]. Our results show that native American dog populations were also impacted. The extent of this impact is unexpected because of the large historical population size of dogs in the Americas and the existence of potential refugia (*e.g*. isolated human groups) where native lineages could have survived. Several factors might have contributed to this replacement, including direct persecution [7,9], preference for the often larger newly arrived dogs, or susceptibility to introduced infectious diseases. Future studies including more ancient and modern dogs and more genetic markers, such as neutral autosomal markers, genes of known function and Y-chromosome markers, will contribute to a deeper understanding of the causes of and extent to which the native American dog population has changed since the arrival of Columbus.

## Conclusions

Using molecular data and statistical modeling, we demonstrate that an important amount of mitochondrial haplotype diversity exists in undersampled non-breed dog populations, and that the breadth of the impact of post-Columbian colonization on the Americas has been underestimated. The extensive replacement of the native American dog population inferred from our data set illustrates that even cultural and biological elements that are not specific targets of invaders can be profoundly affected at a continental scale and in a short period of time.

## Methods

We used published primers (ThrL and DLHc) and PCR conditions [1] to amplify and sequence in both directions a fragment of 425 bp from the mitochondrial control region (Table S1). Sequences were compared to available sequences in GenBank with megablast to check for nuclear insertions and new haplotypes. Additional published sequences from dogs [1,3,4,10] and wolves [10-14] were included to place our study in a historical and geographical framework (*e.g*. to root phylogenetic trees) as in [1-4].

We used the E-INS-i option in MAFFT [15] to align all sequences included in the study. We performed phylogenetic analysis of the complete dataset with and without removing redundant haplotypes using MacClade 4.06 [16]. We ran 1000 searches and 2000 bootstrap replicates in Garli0.96 [17] to search for a maximum likelihood topology under the model TrN+I+G selected by Modeltest 3.7 [18] using the Akaike information criterion. Sequences that clustered in Clade I [3], were further analyzed by constructing haplotype networks. We focused on Clade I because all but one of the ancient American dog sequences cluster within this clade. We built median-joining [19] haplotype networks in NETWORK 4.5 http://www.fluxus-engineering.com/ and a statistical parsimony network in TCS 1.21 [20] with gaps as an informative fifth state. Both methods yielded similar results and only the result of the former analysis is shown.

We estimated mitochondrial effective population size () for the ancient sequences described by reference [1] and a corresponding subset of our modern sequences where all samples collected north of Mexico were excluded in order to better match the geographical range of the ancient dogs from reference [1] that died before Columbus first arrived to the Americas. We used nucleotide diversity *π *as a direct estimate of the population-scaled mutation rate *θ_π _*and the expression *θ_π _*= 2*N_e_μ *where *μ *is the mutation rate per nucleotide and generation (note that *N_e _*will be affected by inclusion of multiple clades). We computed *π *using DNAsp v.5.00.07 [21], and used a conservative estimate of *μ *in the dog mitochondrial control region that assumed a divergence time between gray wolves and coyotes 2 million years ago (2.13 × 10^-7 ^per generation) [4]. We note that assuming a 1 million year divergence time (*e.g*. [22]) would result in higher estimates of  and thus decrease the probability of observing large differences between samples from different time points even further. We also note that mtDNA data is not optimal for accurately estimating  in natural populations, but its use here is motivated by our interest for the possible magnitude of genetic drift acting on American dog mitochondria, particularly in the last ~1000 years (see below), and by the fact that no autosomal sequence data is currently available.

We investigated the probability of observing a private haplogroup in the ancient sample with a frequency as high as Clade I*a *in a hypothesis-testing framework for three different population genetic models for the demographic history of American dogs using Serial Sim-Coal [23,24], the only coalescent simulator currently available that allows modeling of both population structure and samples from different time points. By simulating 10,000 independent genealogical histories and investigating the resulting topologies with custom scripts, we approximated the probability that at least 8 of 13 lineages sampled 1000 years ago (corresponding to the ancient Latin American samples) [1] would be monophyletic with respect to 299 lineages sampled at the present (corresponding to modern Latin American samples included in the study; Figure 3A), given different assumptions about *N_e_*. The general approach of estimating the possible contribution of Native American dogs to the extant gene pool in a simulation-based hypothesis-testing framework was chosen because few analytical tools (*i.e*. mathematical models) have been developed to deal with probabilities under the structured coalescent with temporal samples. Simulations thus provide a flexible alternative.

First, we tested a demographic model with a single continuous population that either maintained a constant size during all of its history or started growing exponentially 200 generations ago to an *N_e _*of 15,000. To investigate the possible contribution of the native American dog population to the current population, we constructed an isolation-admixture model more in agreement with historical evidence (Figure 3B). Starting from a single population at the present, the lineages in the population were divided into two isolated populations 400 years ago (representing the post-Columbian colonization of the Americas by Europeans) with probability *c *and 1-*c*, respectively. At 1000 years ago, 13 new samples were taken from one of the subpopulations (New World, representing the data available on ancient Latin American dogs [1]). At 13,000 years further in the past the American population underwent a bottleneck, implying a tenfold reduction on its effective size, and was joined together again with the Old World population at 14,000 years ago, representing the isolation of American dogs from the Eurasian populations from which they originated [1]. The exact time American and Eurasian populations were isolated is the subject of much controversy, so we use this conservative number (14,000 years before present). However, we found that the timing of this event and the severity of the bottleneck had only marginal effects on the probability of a private ancient haplogroup compared to the admixture proportions. We investigated the effect of assuming a generation time of both 2 and 3 years.

## Authors' contributions

JAL, SC-F and CV conceived and designed the study. SC-F, RV, JAL and CV collected samples. PS and SC-F performed laboratory analyses. SC-F and PS performed sequence analyses and phylogenetic analyses. PS designed and performed population genetic analyses and simulations. SC-F, PS and JAL prepared the manuscript. All authors commented on the results, and read and approved the final manuscript.

## Supplementary Material

Additional file 1 **Table S1: Table describing all samples used, including GenBank number, collector, country of origin and clade to which the haplotypes belongs, as defined in**[3]**and**[4]**.** Click here for file
