# Supplementary material for: Vanishing native American dog lineages
Source: BMC Evol Biol. 2011 Mar 21;11:73. doi: 10.1186/1471-2148-11-73 (PMC3076259; doi:10.1186/1471-2148-11-73)
Supplement: Additional file 1 — Table S1: Table describing all samples used, including GenBank number, collector, country of origin and clade to which the haplotypes belongs, as defined in[3]and[4]. [file 1471-2148-11-73-S1.PDF]

Table S1. Modern American dog samples used in this study. Genbank number of sequence, collector of dog sample and country where sample was collected are listed. Clade refers to the mitochondrial DNA clade as defined in [3] followed by [4] to which the sequence belongs.

Sequence ID	GenBank Number	Collector/Reference	Country	Clade
A2	HQ126702	J. Torres	Argentina	I, A
A3	HQ126703	G. Bazzano	Argentina	I, A
A4	HQ126704	J. Torres	Argentina	I, A
A5	HQ126705	J. Torres	Argentina	I, A
A8	HQ126706	Y. Cuevas	Argentina	I, A
A9	HQ126707	J. Torres	Argentina	IV, B
A11	HQ126708	J. Torres	Argentina	I, A
A12	HQ126709	J. Torres	Argentina	IV, B
A13	HQ126710	J. Torres	Argentina	I, A
A14	HQ126711	J. Torres	Argentina	I, A
A15	HQ126712	J. Torres	Argentina	I, A
A17	HQ126713	J. Torres	Argentina	I, A
A18	HQ126714	J. Torres	Argentina	I, A
A19	HQ126715	J. Torres	Argentina	IV, B
A21	HQ126716	J. Torres	Argentina	I, A
A22	HQ126717	J. Torres	Argentina	I, A
A23	HQ126718	J. Torres	Argentina	I, A
A24	HQ126719	J. Torres	Argentina	II, C
A25	HQ126720	L. Hoyos	Argentina	I, A
A26	HQ126721	J. Ruíz-Garcia	Argentina	II, C
A28	HQ126722	J. Torres	Argentina	IV, B
A29	HQ126723	J. Torres	Argentina	I, A
A30	HQ126724	J. Torres	Argentina	IV, B
A31	HQ126725	J. Torres	Argentina	II, C
A32	HQ126726	J. Torres	Argentina	I, A
A33	HQ126727	J. Torres	Argentina	I, A
A34	HQ126728	J. Torres	Argentina	I, A
A35	HQ126729	J. Torres	Argentina	I, A
A36	HQ126730	J. Torres	Argentina	IV, B
A37	HQ126731	J. Torres	Argentina	I, A
A40	HQ126732	J. Torres	Argentina	I, A
A41	HQ126733	J. Torres	Argentina	I, A
A42	HQ126734	J. Torres	Argentina	I, A
A44	HQ126735	J. Torres	Argentina	I, A
A46	HQ126736	J. Torres	Argentina	I, A
A47	HQ126737	J. Torres	Argentina	I, A

A48	HQ126738	J. Torres	Argentina	IV, B
A49	HQ126739	J. Torres	Argentina	I, A
A50	HQ126740	J. Torres	Argentina	I, A
A51	HQ126741	J. Torres	Argentina	I, A
A52	HQ126742	Y. Cuevas	Argentina	IV, B
A53	HQ126743	J. Torres	Argentina	I, A
A54	HQ126744	C. Sanhueza	Argentina	I, A
B1	HQ126745	A. Koeler	Brazil	I, A
B2	HQ126746	A. Koeler	Brazil	II, C
B3	HQ126747	A. Koeler	Brazil	IV, B
B4	HQ126748	A. Koeler	Brazil	IV, B
B5	HQ126749	A. Koeler	Brazil	I, A
B6	HQ126750	A. Koeler	Brazil	II, C
B7	HQ126751	A. Koeler	Brazil	IV, B
B8	HQ126752	A. Koeler	Brazil	I, A
B9	HQ126753	A. Koeler	Brazil	I, A
B10	HQ126754	A. Koeler	Brazil	IV, B
B11	HQ126755	A. Koeler	Brazil	II, C
B12	HQ126756	A. Koeler	Brazil	IV, B
B14	HQ126757	A. Koeler	Brazil	IV, B
B15	HQ126758	A. Koeler	Brazil	I, A
B16	HQ126759	A. Koeler	Brazil	I, A
B18	HQ126760	A. Koeler	Brazil	I, A
B19	HQ126761	A. Koeler	Brazil	I, A
B20	HQ126762	A. Koeler	Brazil	IV, B
B21	HQ126763	A. Koeler	Brazil	IV, B
B22	HQ126764	A. Koeler	Brazil	I, A
B23	HQ126765	A. Koeler	Brazil	I, A
B24	HQ126766	A. Koeler	Brazil	I, A
B25	HQ126767	A. Koeler	Brazil	I, A
B26	HQ126768	A. Koeler	Brazil	IV, B
B28	HQ126769	A. Koeler	Brazil	I, A
B29	HQ126770	A. Koeler	Brazil	II, C
B30	HQ126771	A. Koeler	Brazil	I, A
B31	HQ126772	A. Koeler	Brazil	I, A
B32	HQ126773	A. Koeler	Brazil	II, C
B33	HQ126774	A. Koeler	Brazil	I, A
B34	HQ126775	A. Koeler	Brazil	I, A
B35	HQ126776	A. Koeler	Brazil	II, C
B36	HQ126777	A. Koeler	Brazil	I, A
B37	HQ126778	A. Koeler	Brazil	I, A
B38	HQ126779	A. Koeler	Brazil	IV, B
B39	HQ126780	A. Koeler	Brazil	IV, B
B41	HQ126781	A. Koeler	Brazil	IV, B

B42	HQ126782	A. Koeler	Brazil	I, A
B43	HQ126783	A. Koeler	Brazil	I, A
B44	HQ126784	A. Koeler	Brazil	IV, B
B45	HQ126785	A. Koeler	Brazil	I, A
B46	HQ126786	A. Koeler	Brazil	I, A
BO1	HQ126787	M. Merchán	Bolivia	IV, B
BO2	HQ126788	M. Merchán	Bolivia	II, C
BO3	HQ126789	M. Merchán	Bolivia	II, C
BO4	HQ126790	M. Merchán	Bolivia	II, C
BO6	HQ126791	M. Merchán	Bolivia	I, A
BO7	HQ126792	M. Merchán	Bolivia	I, A
BO8	HQ126793	M. Merchán	Bolivia	I, A
BO10	HQ126794	V. Mendoza	Bolivia	IV, B
BO11	HQ126795	P. Tinas	Bolivia	II, C
BO12	HQ126796	F. Alejandro	Bolivia	II, C
BO13	HQ126797	D. Guarachi	Bolivia	II, C
BO14	HQ126798	D. Guarachi	Bolivia	II, C
BO15	HQ126799	D. Guarachi	Bolivia	I, A
BO16	HQ126800	D. Guarachi	Bolivia	I, A
BO17	HQ126801	D. Guarachi	Bolivia	II, C
BO18	HQ126802	D. Guarachi	Bolivia	II, C
BO19	HQ126803	D. Guarachi	Bolivia	I, A
BO20	HQ126804	V. Mendoza	Bolivia	IV, B
BO21	HQ126805	P. Torres	Bolivia	IV, B
BO22	HQ126806	P. Torres	Bolivia	II, C
BO23	HQ126807	P. Torres	Bolivia	I, A
BO24	HQ126808	P. Torres	Bolivia	I, A
BO25	HQ126809	Fundación Proteja	Bolivia	III, D
BO26	HQ126810	D. Guarachi	Bolivia	II, C
BO27	HQ126811	E. Donaire	Bolivia	IV, B
BO28	HQ126812	V. Mendoza	Bolivia	I, A
BO29	HQ126813	M. Calisaya	Bolivia	I, A
BZ01	HQ126814	F. Hailer	Belize	I, A
BZ03	HQ126815	F. Hailer	Belize	I, A
BZ04	HQ126816	F. Hailer	Belize	I, A
BZ05	HQ126817	F. Hailer	Belize	I, A
BZ06	HQ126818	F. Hailer	Belize	I, A
BZ07	HQ126819	F. Hailer	Belize	IV, B
C1	HQ126820	Herrera family	Cuba	I, A
C2	HQ126821	Herrera family	Cuba	I, A
C3	HQ126822	Herrera family	Cuba	I, A
C4	HQ126823	Herrera family	Cuba	I, A
C5	HQ126824	Herrera family	Cuba	I, A

CA1	HQ126825	M. Dumond, L. E. Harding	Canada	I, A
CA2	HQ126826	M. Dumond, L. E. Harding	Canada	IV, B
CA4	HQ126827	M. Dumond, L. E. Harding	Canada	I, A
CA5	HQ126828	M. Dumond, L. E. Harding	Canada	IV, B
CA6	HQ126829	M. Dumond, L. E. Harding	Canada	I, A
CA7	HQ126830	M. Dumond, L. E. Harding	Canada	I, A
CA8	HQ126831	M. Dumond, L. E. Harding	Canada	I, A
CA9	HQ126832	M. Dumond, L. E. Harding	Canada	I, A
CA10	HQ126833	D. White, L. E. Harding	Canada	I, A
JAL4799	HQ126834	C. Darimont	Canada	I, A
JAL5173	HQ126835	H. Bryan	Canada	IV, B
JAL5174	HQ126836	H. Bryan	Canada	I, A
JAL5175	HQ126837	H. Bryan	Canada	I, A
JAL5176	HQ126838	H. Bryan	Canada	I, A
JAL5177	HQ126839	H. Bryan	Canada	I, A
JAL5178	HQ126840	H. Bryan	Canada	I, A
JAL5179	HQ126841	H. Bryan	Canada	I, A
JAL5181	HQ126842	H. Bryan	Canada	I, A
JAL5182	HQ126843	H. Bryan	Canada	I, A
JAL5183	HQ126844	H. Bryan	Canada	I, A
JAL5184	HQ126845	H. Bryan	Canada	I, A
JAL5185	HQ126846	H. Bryan	Canada	IV, B
JAL5186	HQ126847	H. Bryan	Canada	I, A
JAL5187	HQ126848	H. Bryan	Canada	I, A
JAL5188	HQ126849	H. Bryan	Canada	IV, B
JAL5189	HQ126850	H. Bryan	Canada	I, A
JAL5190	HQ126851	H. Bryan	Canada	IV, B
JAL5191	HQ126852	H. Bryan	Canada	I, A
JAL5192	HQ126853	H. Bryan	Canada	I, A
JAL5193	HQ126854	H. Bryan	Canada	IV, B
JAL5194	HQ126855	H. Bryan	Canada	I, A
JAL5196	HQ126856	H. Bryan	Canada	I, A
JAL5197	HQ126857	H. Bryan	Canada	I, A
JAL5198	FN298190	[7]	Canada	I, A
JAL5199	FN298191	[7]	Canada	I, A
JAL5200	FN298192	[7]	Canada	IV, B
JAL5201	FN298193	[7]	Canada	I, A
JAL5202	FN298194	[7]	Canada	I, A
JAL5203	FN298195	[7]	Canada	I, A
JAL5204	FN298196	[7]	Canada	IV, B
JAL5205	HQ126858	H. Bryan	Canada	I, A
JAL5206	FN298197	[7]	Canada	I, A
JAL5207	HQ126859	H. Bryan	Canada	I, A
JAL5208	FN298198	[7]	Canada	I, A

JAL5209	FN298199	[7]	Canada	I, A
JAL5210	FN298200	[7]	Canada	I, A
JAL5211	FN298201	[7]	Canada	I, A
JAL5212	FN298202	[7]	Canada	I, A
JAL5213	FN298203	[7]	Canada	I, A
JAL5214	FN298204	[7]	Canada	I, A
JAL5215	FN298205	[7]	Canada	IV, B
JAL5216	FN298206	[7]	Canada	I, A
JAL5217	FN298207	[7]	Canada	I, A
JAL5218	FN298208	[7]	Canada	I, A
JAL5219	FN298209	[7]	Canada	I, A
JAL5220	FN298210	[7]	Canada	IV, B
JAL5221	FN298211	[7]	Canada	II, C
JAL5222	FN298212	[7]	Canada	IV, B
JAL5223	FN298213	[7]	Canada	I, A
JAL5224	FN298214	[7]	Canada	II, C
JAL5225	FN298215	[7]	Canada	I, A
JAL5226	FN298216	[7]	Canada	I, A
JAL5227	FN298217	[7]	Canada	I, A
JAL5228	FN298218	[7]	Canada	IV, B
JAL5230	HQ126860	H. Bryan	Canada	IV, B
JAL5231	HQ126861	H. Bryan	Canada	II, C
JAL5232	HQ126862	H. Bryan	Canada	IV, B
JAL5233	HQ126863	H. Bryan	Canada	I, A
JAL5234	HQ126864	H. Bryan	Canada	I, A
JAL5235	HQ126865	H. Bryan	Canada	I, A
JAL5236	HQ126866	H. Bryan	Canada	I, A
JAL5237	HQ126867	H. Bryan	Canada	I, A
JAL5238	HQ126868	H. Bryan	Canada	I, A
JAL5239	HQ126869	H. Bryan	Canada	IV, B
JAL5240	HQ126870	H. Bryan	Canada	I, A
JAL5241	HQ126871	H. Bryan	Canada	I, A
JAL5242	HQ126872	H. Bryan	Canada	I, A
JAL5243	HQ126873	H. Bryan	Canada	I, A
JAL5244	HQ126874	H. Bryan	Canada	IV, B
JAL5245	HQ126875	H. Bryan	Canada	I, A
JAL5246	HQ126876	H. Bryan	Canada	I, A
JAL5247	HQ126877	H. Bryan	Canada	I, A
JAL5248	HQ126878	H. Bryan	Canada	I, A
JAL5249	HQ126879	H. Bryan	Canada	I, A
JAL5250	HQ126880	H. Bryan	Canada	I, A
JAL5251	HQ126881	H. Bryan	Canada	I, A
JAL5257	HQ126882	H. Bryan	Canada	I, A
JAL5258	HQ126883	H. Bryan	Canada	I, A

JAL5260	HQ126884	H. Bryan	Canada	I, A
JAL5262	HQ126885	H. Bryan	Canada	IV, B
JAL5263	HQ126886	H. Bryan	Canada	IV, B
JAL5264	HQ126887	H. Bryan	Canada	IV, B
JAL5265	HQ126888	H. Bryan	Canada	IV, B
JAL5266	HQ126889	H. Bryan	Canada	IV, B
JAL5267	HQ126890	H. Bryan	Canada	I, A
JAL5268	HQ126891	H. Bryan	Canada	IV, B
JAL5269	HQ126892	H. Bryan	Canada	I, A
JAL5271	HQ126893	H. Bryan	Canada	I, A
JAL5272	HQ126894	H. Bryan	Canada	I, A
JAL5273	HQ126895	H. Bryan	Canada	I, A
JAL5274	HQ126896	H. Bryan	Canada	IV, B
JAL5275	HQ126897	S. Kauffmann	Canada	I, A
JAL5276	HQ126898	S. Kauffmann	Canada	I, A
CR59	HQ126899	I. Menche, A. Quintana	Costa Rica	I, A
CR60	HQ126900	I. Menche, A. Quintana	Costa Rica	I, A
CR61	HQ126901	I. Menche, A. Quintana	Costa Rica	I, A
M1	HQ126902	A. Alonso	Mexico	I, A
M4	HQ126903	R. Telly	Mexico	IV, B
M5	HQ126904	R. Telly	Mexico	IV, B
M6	HQ126905	R. Telly	Mexico	I, A
M7	HQ126906	R. Telly	Mexico	I, A
M8	HQ126907	R. Telly	Mexico	I, A
M9	HQ126908	R. Telly	Mexico	I, A
M10	HQ126909	R. Telly	Mexico	I, A
M11	HQ126910	R. Telly	Mexico	I, A
M12	HQ126911	R. Telly	Mexico	I, A
M13	HQ126912	N. Estrada	Mexico	IV, B
M14	HQ126913	N. Estrada	Mexico	I, A
M15	HQ126914	N. Estrada	Mexico	I, A
M16	HQ126915	N. Estrada	Mexico	I, A
M17	HQ126916	N. Estrada	Mexico	I, A
M18	HQ126917	N. Estrada	Mexico	I, A
M19	HQ126918	N. Estrada	Mexico	I, A
M21	HQ126919	N. Estrada	Mexico	I, A
M24	HQ126920	R. Telly	Mexico	IV, B
M25	HQ126921	R. Telly	Mexico	I, A
M26	HQ126922	R. Telly	Mexico	I, A
M27	HQ126923	R. Valadéz, C. Álvarez	Mexico	I, A
M28	HQ126924	R. Valadéz, C. Álvarez	Mexico	I, A
M29	HQ126925	R. Valadéz, C. Álvarez	Mexico	I, A
M30	HQ126926	R. Valadéz, C. Álvarez	Mexico	I, A

M31	HQ126927	R. Valadéz, C. Álvarez	Mexico	IV, B
M32	HQ126928	R. Valadéz, C. Álvarez	Mexico	I, A
M33	HQ126929	R. Valadéz, C. Álvarez	Mexico	IV, B
M35	HQ126930	R. Valadéz, C. Álvarez	Mexico	IV, B
M36	HQ126931	R. Valadéz, C. Álvarez	Mexico	I, A
M37	HQ126932	R. Valadéz, C. Álvarez	Mexico	I, A
M38	HQ126933	R. Valadéz, C. Álvarez	Mexico	I, A
M39	HQ126934	R. Valadéz, C. Álvarez	Mexico	I, A
M40	HQ126935	R. Valadéz, C. Álvarez	Mexico	I, A
M41	HQ126936	R. Valadéz, C. Álvarez	Mexico	I, A
M42	HQ126937	B. Rodríguez	Mexico	I, A
M43	HQ126938	B. Rodríguez	Mexico	I, A
M44	HQ126939	B. Rodríguez	Mexico	I, A
M45	HQ126940	Mestre	Mexico	I, A
M45b	HQ126941	Mestre	Mexico	I, A
M46	HQ126942	Mestre	Mexico	I, A
M47	HQ126943	Mestre	Mexico	IV, B
M48	HQ126944	Mestre	Mexico	IV, B
M49	HQ126945	Mestre	Mexico	I, A
M50	HQ126946	R. Valadéz	Mexico	I, A
M53	HQ126947	R. Valadéz	Mexico	I, A
M54	HQ126948	R. Valadéz	Mexico	IV, B
M55a	HQ126949	R. Valadéz	Mexico	I, A
M55b	HQ126950	R. Valadéz	Mexico	I, A
M56	HQ126951	R. Valadéz	Mexico	IV, B
M57	HQ126952	R. Valadéz	Mexico	IV, B
M59	HQ126953	R. Valadéz	Mexico	I, A
M61	HQ126954	R. Valadéz	Mexico	IV, B
M62	HQ126955	R. Valadéz	Mexico	IV, B
M63	HQ126956	R. Valadéz	Mexico	I, A
M64	HQ126957	R. Valadéz	Mexico	I, A
M65	HQ126958	R. Valadéz	Mexico	IV, B
M67	HQ126959	R. Valadéz	Mexico	I, A
M68	HQ126960	R. Valadéz	Mexico	II, C
M69	HQ126961	R. Valadéz	Mexico	II, C
M70	HQ126962	R. Valadéz	Mexico	I, A
M71	HQ126963	R. Valadéz	Mexico	II, C
M73	HQ126964	R. Valadéz	Mexico	IV, B
M74	HQ126965	R. Valadéz	Mexico	II, C
M75	HQ126966	R. Valadéz	Mexico	I, A
M76	HQ126967	R. Valadéz	Mexico	I, A
M77	HQ126968	R. Valadéz	Mexico	IV, B
MD_3.1	HQ126969	J. Leonard	Mexico	I, A
MD_3.2	HQ126970	J. Leonard	Mexico	IV, B

MD_3.3	HQ126971	J. Leonard	Mexico	I, A
MD_3.4	HQ126972	J. Leonard	Mexico	I, A
MD 3.5	HQ126973	J. Leonard	Mexico	II, C
P2	HQ126974	J. Castroviejo	Paraguay	I, A
PA1	HQ126975	N. Bastidas	Panama	II, C
PA2	HQ126976	N. Bastidas	Panama	IV, B
PA3	HQ126977	N. Bastidas	Panama	IV, B
PA6	HQ126978	N. Bastidas	Panama	IV, B
PA7	HQ126979	N. Bastidas	Panama	I, A
PA8	HQ126980	N. Bastidas	Panama	I, A
PA11	HQ126981	A. Ibáñez	Panama	I, A
PA13	HQ126982	O. Rodríguez	Panama	IV, B
PA14	HQ126983	N. Bastidas	Panama	I, A
PA16	HQ126984	O. Rodríguez	Panama	IV, B
PE1	HQ126985	S. Castroviejo	Peru	I, A
PE2	HQ126986	S. Castroviejo	Peru	II, C
PE4	HQ126987	S. Castroviejo	Peru	II, C
PE6	HQ126988	S. Castroviejo	Peru	I, A
PE7	HQ126989	S. Castroviejo	Peru	IV, B
PE8	HQ126990	S. Castroviejo	Peru	IV, B
PE9	HQ126991	S. Castroviejo	Peru	IV, B
PE10	HQ126992	S. Castroviejo	Peru	II, C
PE11	HQ126993	S. Castroviejo	Peru	III, D
PE12	HQ126994	S. Castroviejo	Peru	II, C
PE13	HQ126995	S. Castroviejo	Peru	I, A
PE15	HQ126996	S. Castroviejo	Peru	IV, B
PE16	HQ126997	S. Castroviejo	Peru	I, A
PE17	HQ126998	S. Castroviejo	Peru	I, A
PE18	HQ126999	S. Castroviejo	Peru	IV, B
PE20	HQ127000	S. Castroviejo	Peru	I, A
PE21	HQ127001	S. Castroviejo	Peru	I, A
PE22	HQ127002	S. Castroviejo	Peru	II, C
PE23	HQ127003	S. Castroviejo	Peru	I, A
PE24	HQ127004	S. Castroviejo	Peru	IV, B
PE25	HQ127005	S. Castroviejo	Peru	IV, B
PE26	HQ127006	S. Castroviejo	Peru	I, A
PE27	HQ127007	S. Castroviejo	Peru	IV, B
U1	HQ127008	J. Leonard	USA	IV, B
U2	HQ127009	J. Leonard	USA	IV, B
UR1	HQ127010	V. Calvo	Uruguay	I, A
UR2	HQ127011	V. Calvo	Uruguay	I, A
UR3	HQ127012	V. Calvo	Uruguay	I, A
UR4	HQ127013	V. Calvo	Uruguay	I, A

UR6	HQ127014	V. Calvo	Uruguay	I, A
UR8	HQ127015	V. Calvo	Uruguay	I, A
V1	HQ127016	S. Castroviejo	Venezuela	I, A
V2	HQ127017	S. Castroviejo	Venezuela	I, A
V3	HQ127018	S. Castroviejo	Venezuela	IV, B
V3b	HQ127019	S. Castroviejo	Venezuela	IV, B
V4	HQ127020	S. Castroviejo	Venezuela	I, A
V5	HQ127021	S. Castroviejo	Venezuela	I, A
V6	HQ127022	S. Castroviejo	Venezuela	I, A
V7	HQ127023	S. Castroviejo	Venezuela	II, C
V8	HQ127024	S. Castroviejo	Venezuela	I, A
V11	HQ127025	S. Castroviejo	Venezuela	I, A
V12	HQ127026	S. Castroviejo	Venezuela	I, A
V13	HQ127027	J. Ruíz, R. Antelo	Venezuela	I, A
V14	HQ127028	J. Ruíz, R. Antelo	Venezuela	I, A
V16	HQ127029	J. Ruíz, R. Antelo	Venezuela	I, A
V17	HQ127030	J. Ruíz, R. Antelo	Venezuela	I, A
V18	HQ127031	J. Ruíz, R. Antelo	Venezuela	I, A
V19	HQ127032	J. Ruíz, R. Antelo	Venezuela	I, A
V20	HQ127033	J. Ruíz, R. Antelo	Venezuela	IV, B
V21	HQ127034	J. Ruíz, R. Antelo	Venezuela	I, A
V22	HQ127035	J. Ruíz, R. Antelo	Venezuela	I, A
V23	HQ127036	J. Ruíz, R. Antelo	Venezuela	I, A
V25	HQ127037	J. Ruíz, R. Antelo	Venezuela	I, A
V26	HQ127038	S. Castroviejo	Venezuela	I, A
V27	HQ127039	S. Castroviejo	Venezuela	IV, B
V28	HQ127040	S. Castroviejo	Venezuela	IV, B
V30	HQ127041	S. Castroviejo	Venezuela	I, A
V32	HQ127042	S.Castroviejo, J.M. Castroviejo	Venezuela	I, A
V33	HQ127043	S.Castroviejo, J.M. Castroviejo	Venezuela	IV, B
V34	HQ127044	S.Castroviejo, J.M. Castroviejo	Venezuela	IV, B
V35	HQ127045	S.Castroviejo, J.M. Castroviejo	Venezuela	I, A
V36	HQ127046	S.Castroviejo, J.M. Castroviejo	Venezuela	I, A
V37	HQ127047	S.Castroviejo, J.M. Castroviejo	Venezuela	I, A
V38	HQ127048	S.Castroviejo, J.M. Castroviejo	Venezuela	I, A
V45	HQ127049	S.Castroviejo, J.M. Castroviejo	Venezuela	I, A
V48	HQ127050	S.Castroviejo, J.M. Castroviejo	Venezuela	I, A
V49	HQ127051	S.Castroviejo, J.M. Castroviejo	Venezuela	I, A
V51	HQ127052	C.Vila, S.Castroviejo	Venezuela	I, A
V52	HQ127053	C.Vila, S.Castroviejo	Venezuela	I, A
V53	HQ127054	C.Vila, S.Castroviejo	Venezuela	IV, B
V54	HQ127055	C.Vila, S.Castroviejo	Venezuela	II, C
V55	HQ127056	C.Vila, S.Castroviejo	Venezuela	IV, B
V56	HQ127057	C.Vila, S.Castroviejo	Venezuela	IV, B

V58	HQ127058	C.Vila, S.Castroviejo	Venezuela	IV, B
V59	HQ127059	R. Antelo	Venezuela	II, C
V60	HQ127060	R. Antelo	Venezuela	II, C
V61	HQ127061	R. Antelo	Venezuela	II, C
V62	HQ127062	R. Antelo	Venezuela	I, A
V63	HQ127063	R. Antelo	Venezuela	IV, B
V64	HQ127064	R. Antelo	Venezuela	IV, B
V65	HQ127065	R. Antelo	Venezuela	I, A
V66	HQ127066	R. Antelo	Venezuela	IV, B
V67	HQ127067	R. Antelo	Venezuela	I, A
V68	HQ127068	R. Antelo	Venezuela	I, A
V69	HQ127069	R. Antelo	Venezuela	I, A
V70	HQ127070	R. Antelo	Venezuela	I, A
V71	HQ127071	R. Antelo	Venezuela	IV, B
V72	HQ127072	R. Antelo	Venezuela	II, C
